# In situ reconstruction of ruptured mycotic iliac artery aneurysm with autologous fascial-peritoneal tissue: a case report and literature review

**DOI:** 10.1186/s12893-022-01523-0

**Published:** 2022-02-26

**Authors:** GuiFeng Sang, XiaoYan Guo, GuoLong Liu, HaiJie Che

**Affiliations:** Department of Vascular Surgery, YanTai Yuhuangding Hospital, No. 20, Yuhuangding East Road, YanTai, ShanDong 264000 China

**Keywords:** Infectious pseudoaneurysm, Fascial-peritoneal tissue, Surgery, Case report

## Abstract

**Background:**

Infectious aneurysms are rare in clinic with poor therapeutic outcomes. When artery rupture occurs, the disease tends to progress resulting in a high mortality, and there remains no ideal treatment.

**Case presentation:**

We report a case of rupture of infectious iliac artery pseudoaneurysm, who was assigned to receive artery reconstruction with autologous fascial-peritoneal tissue and obtained satisfied short-term outcome. The follow-up of 6 months after operation was good and long-term follow-up is continuing.

**Conclusion:**

The posterior rectus fascia-peritoneal layer seems to be a feasible autologous biomaterial for vascular substitution in urgent setting when no other autologous material was available.

## Background

Infectious aneurysm is a result of bacterial invasion or autogenous vascular and blood infections. It has a low incidence and occurs in ∼ 0.5–2.0% of aneurysms [1]. In contrast, infectious aneurysm is associated with high mortality due to the high incidence of rupture of the arterial wall damaged by bacterial invasion. Patients with some degree of immunosuppression such as diabetes, chronic renal failure or chronic steroid use, are susceptible to infectious aneurysm [1]. The most common responsible organism of infected aortic aneurysm was Staphylococcus aureus and Salmonella sp. [2] Surgery remains a necessity in this context, as drugs fail to control disease progression and prevent further rupture. Unfortunately, there is no consensus on the surgical approach used [3]. Here, we report a case of infectious iliac artery pseudoaneurysm rupture in a patient who was assigned to receive artery reconstruction with autologous fascial-peritoneal tissue due to the absence of others autologous conduits. To our knowledge, this kind of case has not been reported in the literature.

## Case presentation

**Fig. 1:**
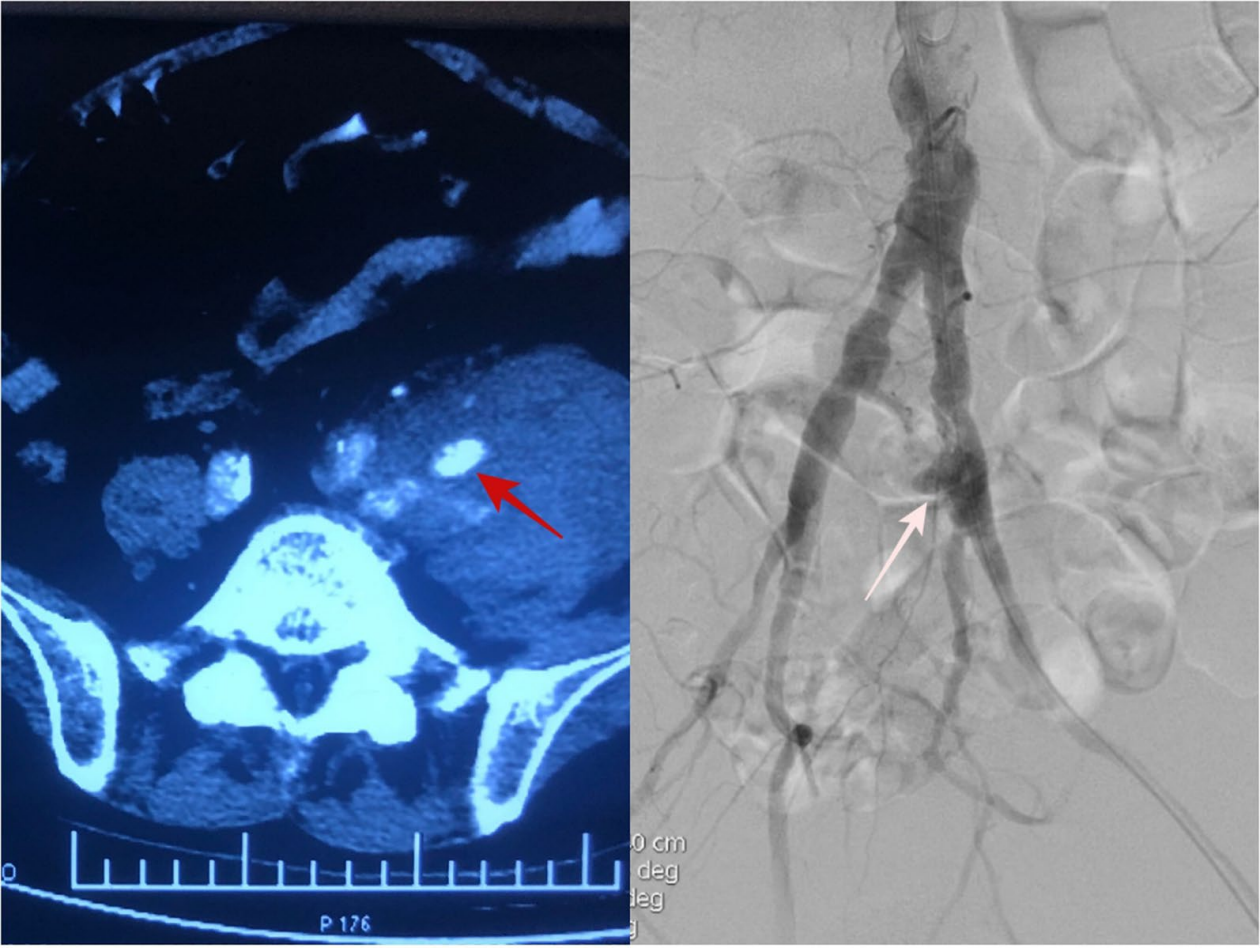
Red arrow: enhanced CT showed hematoma around the left iliac artery and contrast medium extravasation. White arrow: the angiography of the abdominal aorta showed localized outward bulging of the left lower common iliac artery and sign of contrast medium extravasation

**Fig. 2:**
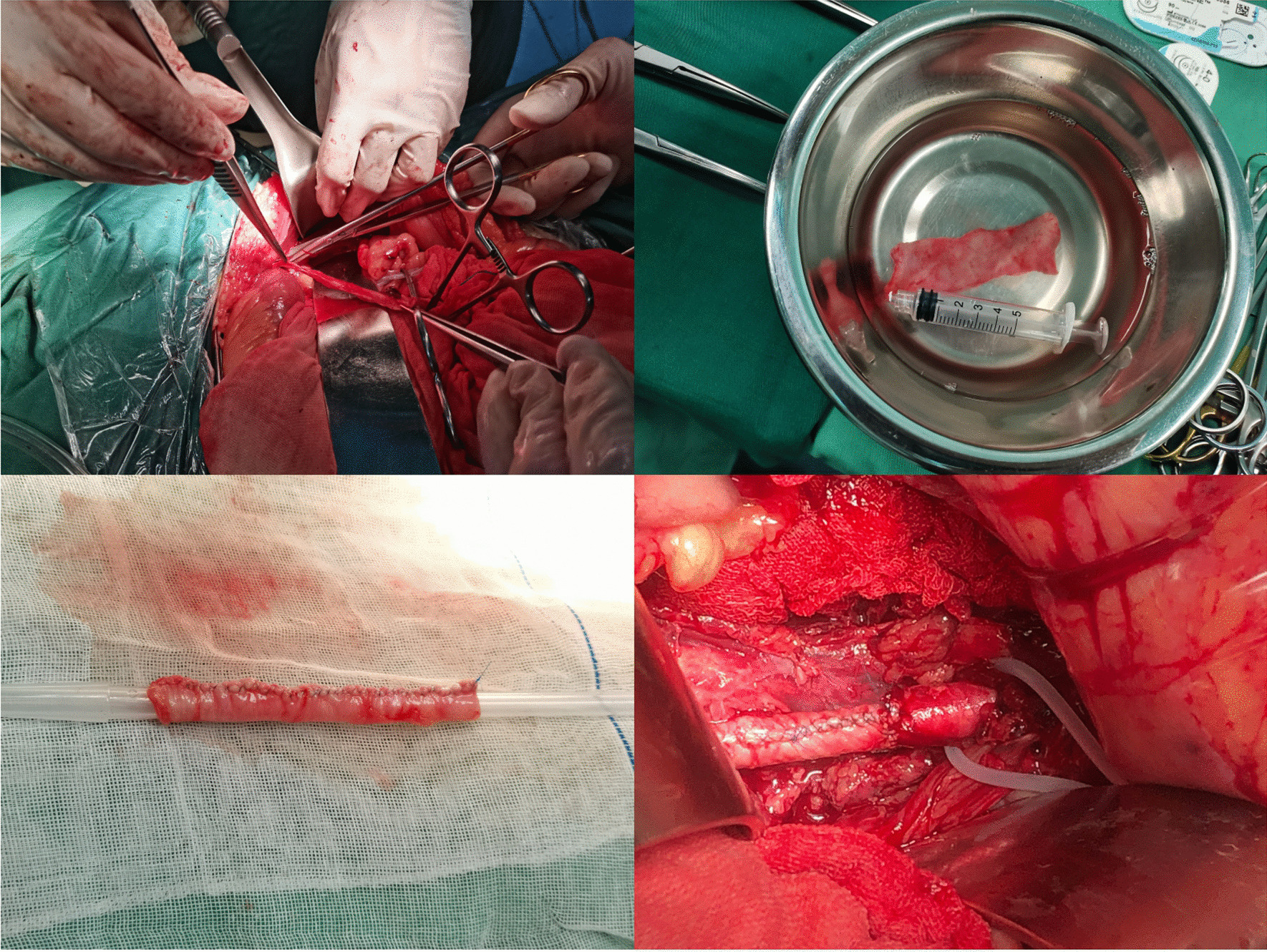
Artery reconstruction was performed to the left iliac common artery and the external iliac artery with a fascia-peritoneum patch based on the arterial caliber

**Fig. 3:**
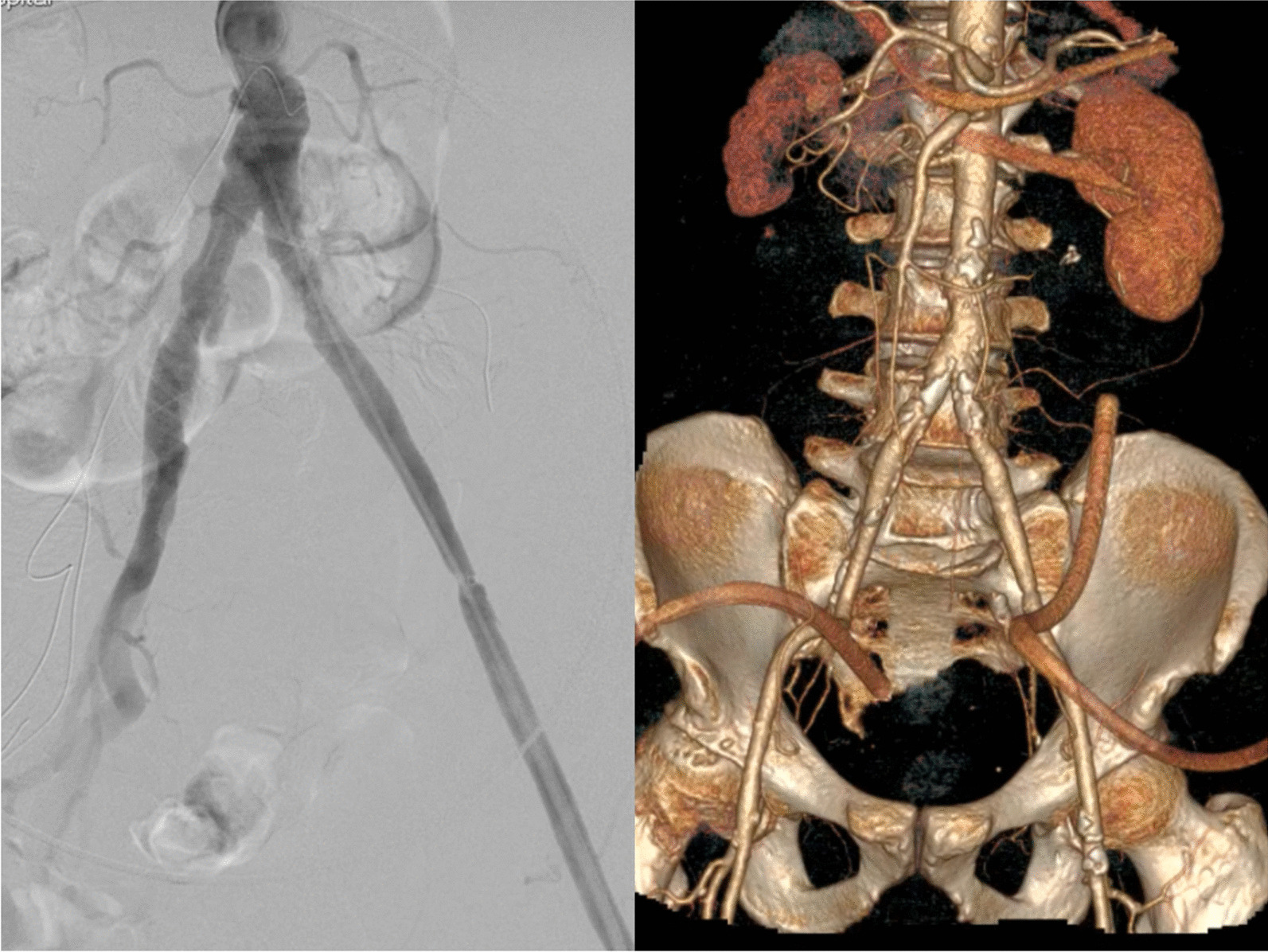
Intraoperative angiography and 1-month CT scan showing patency of the graft in absence of stenosis or dilatation

A 77-year-old male was transferred to our hospital for chills, a high fever for 2 days and left-sided lumbosacral pain for 5 h. The patient had a history of varicose vein surgery on his bilateral great saphenous veins and cholecystectomy for pyogenic cholecystitis. On admission the patient has fever (Temperature 38.2 °C), heart rate 112 beat/min, blood pressure 92/60 mmHg and respiratory rate of 27 breaths/min. Physical examination shows clear consciousness, a feeling of pain, a palpable mass in the left lower abdomen that was pulseless and had marked tenderness, and good bilateral femoral arterial pulses. Routine blood tests showed a White Blood Cell (WBC) count of 19.4 × 10^9^/L, neutrophil percentage of 96.6%, Procalcitonin (PCT) 6.16 ng/mL, Blood Lactate 2.7 mmol/L and C-reactive Protein (CRP) level of 108 mg/L. On enhanced Computed Tomography (CT), manifestations were the occurrence of haematoma around the left iliac artery and contrast medium extravasation (Fig. 1). Rupture of the left iliac artery pseudoaneurysm was diagnosed. The patient was in critical medical condition and was assigned to receive emergency surgery. The patient gave consent for treatment and publication of his clinical notes. The procedure was performed under general anesthesia in an operating theatre equipped with a portable fluoroscopy unit in order to perform and endovascular balloon occlusion for hemorrhage control. After systemic heparinization, percutaneous access was achieved via percutaneous access of left common femoral artery. Abdominal aortography showed localized outward bulging of the left lower common iliac artery and signs of contrast medium extravasation (Fig. 1). Considering the existing typical symptoms of infection and high levels of inflammatory indices in laboratory tests, the patient was suspected to have infectious pseudoaneurysm rupture and was not suitable to receive covered stent implantation. The left common iliac artery block was obtained by balloon introduction, and laparotomy was performed to present visible rupture of the medial wall anterior to the left common iliac artery surrounded by a large haematoma. We excised an 10 × 4 cm segment of posterior rectus fascia-peritoneal layer which lateral to the linea alba and posterior to the left rectus abdominus muscle. Then we used running suture to sew the material into a 7 mm diameter tube with 5−0 Polypropylene non-absorbable suture (PROLENE™, ETHICON, USA) (Fig. 2). Artery reconstruction was then performed on the left common iliac artery and the external iliac artery with a section of the peritoneum and the posterior rectus sheath based on the arterial calibre. Both arteries exhibited good morphology on the aortogram (Fig. 2). Suture of the left internal iliac artery was performed starting from the initial segment. Following haematoma removal, full drainage of hematic and gas was obtained by two indwelling drainage tubes placed in the residual cavity. Local tissues from the arterial wall were harvested for bacterial culture to grow *Enterococcus faecium*. After the operation, he was given meropenem 1.0 g intravenous q12h infusion for 5 days, and then replaced with cefoperazone and sulbactam 2.0 g intravenous infusion q12h infusion for 10 days. Amoxicillin and clavulanate potassium were taken orally for 3 months after discharge. On the CT scan performed 4 weeks after surgery, the left iliac artery and the autologous interposition displayed good morphology, no signs of abscess or hemathoma were present and the indwelling drainage tubes were removed (Fig. 3). The indwelling drainage tubes were pulled out. At 6 months follow-up the patient was in good general condition with no evidence of abdominal wall hernia, fever, abdominal pain, limb pain or intermittent lower limb claudication. Arterial color Doppler ultrasound showed normal blood flow in the left iliac artery.

## Discussion and conclusions

Mycotic aneurysms are a life-threatening condition associated with high mortality rate. Typical clinical symptoms include sepsis, pain in the aneurysm site, and some positive blood tests. Manifestations on CT images are generally irregular arterial walls, rapid aneurysm enlargement and a saccular appearance [4]. Typical surgical approaches are aneurysmectomy resection, clearance of the infection foci and graft interposition. However, surgical treatment of mycotic aneurysm are related to high risk for perioperative mortality (11–21%), amputation (9–11%) and reinfection (3%).^5^Endovascular repair is relatively effective in the context of patients not tolerating the trauma of open surgery. However, artificial material implantation in the infected area is a strategy that goes against the basic principle of surgery and is intensely controversial. It has been reported that open repair with biological grafts is a reliable option for infectious abdominal aneurysms, with excellent anti-infectious effects and favourable overall survival [6]. Heinola et al. showed survival rates of 83% and 71% at 1 year and 5 years, respectively, after the use of biological grafts [7]. Current available biological or autologous materials include bovine pericardial patches, cryopreserved or fresh allograft arteries, autologous superficial femoral veins and jugular veins, which are sometimes not easy to obtain [8, 9].

In the study by Sarac et al., autologous fascial-peritoneal tissue was extracted to repair the aortic stump in a case of abdominal aortic graft infection, which successfully avoided stump rupture and occlusion of the renal artery. The fascia is composed of collagen. Embryologically, the peritoneum and fascia are separately derived from cells similar to those on the endothelial and epithelial layers of blood vessels. Essentially, the rectus fascia-peritonel layer is robust and can be used as an autologous material for aorta repair [10].

In the present case, the patient was transferred to our hospital for iliac artery aneurysm rupture and required emergency surgery. Given that he had a history of varicose vein surgery on his bilateral great saphenous veins, the superficial femoral vein was not available for repair of the rupture. In addition, other allogeneic biomaterials could not be obtained in the case of an emergency. In this context, iliac artery reconstruction was performed with a section of autologous fascial-peritoneal tissue and a good therapeutic outcome was achieved. During the half-year follow-up, there was no severe stenosis or dilatation in the reconstructed section and no occurrence of abdominal wall hernias.

Compared with artificial materials such as dacron prosthesis, autologous tissue has stronger anti-infection ability. The use of posterior rectus fascia-peritoneal layer seems to be a feasible and useful option for treating mycotic aneurysm in urgent setting when no other autologous material was available. However, the inability to obtain large-scale materials limits the size of the reconstructed arteries and lengthier follow-up and large-scale clinical evidence are required to validate the feasibility of such strategy.

## Data Availability

Not applicable.

## Abbreviations

WBC: White blood cell
CRP: C-reactive protein
PCT: Procalcitonin
CT: Computed tomography
